# Supplementary material for: Exploiting the Nucleic Acid Nature of Aptamers for Signal Amplification
Source: Biosensors (Basel). 2022 Nov 4;12(11):972. doi: 10.3390/bios12110972 (PMC9688535; doi:10.3390/bios12110972)
Supplement: Supplementary file 1 [file biosensors-12-00972-s001.zip › biosensors-1982009-supplementary.pdf]

Supplementary Materials

Exploiting the Nucleic Acid Nature of Aptamers for Signal Amplification

Miriam Jauset-Rubio [1,*], Mayreli Ortiz [1] and Ciara K. O'Sullivan [1,2,*]

[1] INTERFIBIO Consolidated Research Group, Department of Chemical Engineering, Universitat Rovira I Virgili, 43007 Tarragona, Spain
[2] Institució Catalana de Recerca I Estudis Avançats (ICREA), Passeig Lluís Companys 23, 08010 Barcelona, Spain
* Correspondence: miriam.jauset@urv.cat (M.J.-R.); ciara.osullivan@urv.cat (C.K.O.)

Table of Contents

Table S1. Sequences used in this work. 3

Figure S1. Cyclic voltammetry performed before and after the surface functionalisation: (a) Carbon screen-printed electrodes; (b) Gold screen-printed electrodes. Cyclic voltammograms were recorded in 1 mM $K_3[Fe(CN)_6]$ and 100 mM KCl at 100 mV/s. 3

Figure S2. Binding studies by Surface Plasmon Resonance (SPR): (a) Calculation of the dissociation constant (K_D) of the unmodified aptamer; (b) Calculation of the dissociation constant (K_D) of the modified biotinylated dUTPs aptamer. The aptamers were prepared in binding buffer (10 mM Phosphate buffer, 135 mM NaCl, 2.5 mM KCl, 1.5 mM $MgCl_2$ pH 7.4) ranging from 125 nM to 0 nM using 1 in 2 dilutions. The binding affinity of the aptamers was determined by BIAevaluation software using a 1:1 Langmuir binding model. The signal of the specific flow cell (with immobilised β-conglutin) was corrected by the subtraction of the signal from the control flow cell (activated with EDC/NHS and blocked with ethanolamine, but without β-conglutin immobilisation). The signals were also corrected via subtraction of the buffer signal obtained. 4

Figure S3. β-conglutin coating optimisation. Different concentrations of immobilised β-conglutin (80 – 0 µg mL^{-1}) in 50 mM carbonate-bicarbonate buffer pH 9.6 were incubated with a constant amount of biotinylated aptamer (1 nM) prepared in binding buffer (10 mM Phosphate buffer, 135 mM NaCl, 2.5 mM KCl, 1.5 mM $MgCl_2$ pH 7.4) followed by the addition of SA-polyHRP (dil. 1 in 20000 of 1 mg mL^{-1}). The absorbances were read at 450 nm after the wells being stopped with 1 M H_2SO_4 5 min later of the TMB addition.5

Figure S4. Optimal amount of modified aptamer. Different concentrations of aptamer (50 – 0 nM) were added to the wells of a microtitre plate modified with 20 µg mL^{-1} of β-conglutin (biotinylated dUTPs aptamer). The supernatant containing any unbound

aptamer was removed and added to a fresh plate, also modified with 20 µg mL^{-1} of β-conglutin (biotinylated dUTPs aptamer supernatant). By comparing both binding curves, 0.4 nM aptamer was demonstrated to be optimal amount of aptamer to use in the competition assay. 5

Figure S5. Evaluation of functionalised carbon screen-printed electrodes using fast chronoamperometry: (a) Signal obtained using fast chronoamperometry from 0 to 0.5 s; (b) Bar graph plotting the signal obtained at 0.5 s. All the aptamers (biotinylated dUTPs aptamer or non cognate aptamer with biotinylated dUTPs) were prepared in binding buffer with a final concentration of 0.4 nM. As controls were used: no coating (without the immobilisation of the target on the surface of the electrode), buffer instead of the aptamer, a cognate aptamer, and three different non cognate targets.**Error! Bookmark not defined.**

Figure S6. Evaluation of functionalised gold screen-printed electrodes using fast chronoamperometry: (a) Signal obtained using fast chronoamperometry from 0 to 0.5 s; (b) Bar graph plotting the signal obtained at 0.5 s. All the aptamers (biotinylated dUTPs aptamer or non cognate aptamer with biotinylated dUTPs) were prepared in binding buffer with a final concentration of 0.4 nM. As controls were used: no coating (without the immobilisation of the target on the surface of the electrode), buffer instead of the aptamer, a cognate aptamer, and three different non cognate targets.**Error! Bookmark not defined.**

Table S1. Sequences used in this work.

Name	Sequence
Forward primer	5'-AGCTCCAGAAGATAAATTACAGG-3'
Reverse phosphorylated primer	5'-PO-GGGGTCATAGTATCCTAGTTG-3'
Aptamer (β-CBA II)	5'- AGCTCCAGAAGATAAATTACAGGGGCCGGGGTGGCTCAGGCAAGGGGTTGACCTGTC GTAGGGATTGTTTTAACAACTAGGATACTATGACCCC-3'
Biotinylated aptamer (β-CBA II)	5'-Biotin- AGCTCCAGAAGATAAATTACAGGGGCCGGGGTGGCTCAGGCAAGGGGTTGACCTGTC GTAGGGATTGTTTTAACAACTAGGATACTATGACCCC-3'
Non cognate aptamer (Seq. 5)	5'- AGCTCCAGAAGATAAATTACAGGTCACGCTCCGCGTACGGTGGGCCGGCGAGGGAAT AGCGGCGCGCGAAAATCAACTAGGATACTATGACCCC-3

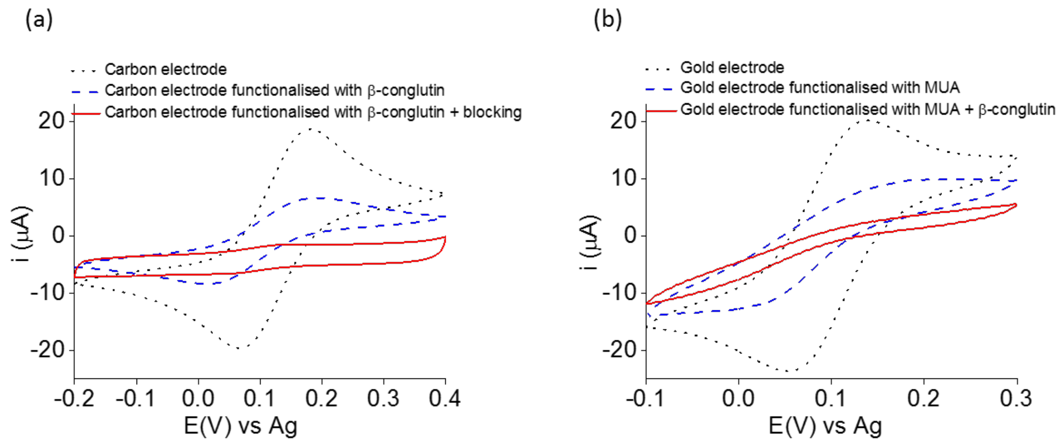

Figure S1. Cyclic voltammetry performed before and after the surface functionalisation: (a) Carbon screen-printed electrodes; (b) Gold screen-printed electrodes. Cyclic voltammograms were recorded in 1 mM $K_3[Fe(CN)_6]$ and 100 mM KCl at 100 mV/s.

(a)

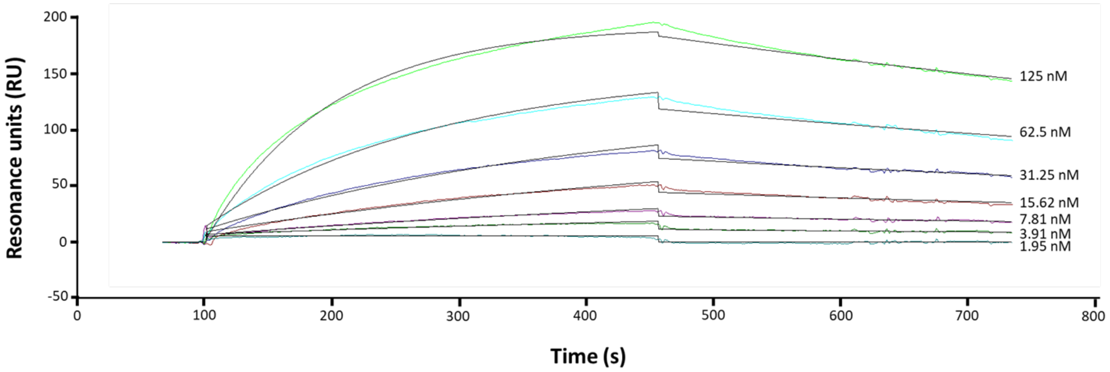

Aptamer	Ka (1 Ms^{-1})	Kd (1 s^{-1})	KD (M)	Chi^2
Unmodified aptamer	$7.5x10^4$	$8.32x10^{-4}$	$1.11x10^{-8}$	3.43

(b)

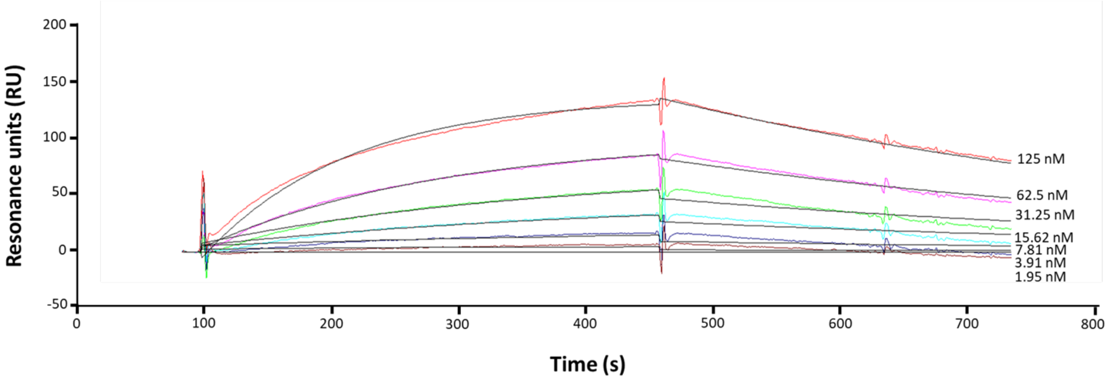

Aptamer	Ka (1 Ms^{-1})	Kd (1 s^{-1})	KD (M)	Chi^2
Modified biotinylated dUTPs aptamer	$1.08x10^5$	$2x10^{-3}$	$1.85x10^{-8}$	6.16

Figure S2. Binding studies by Surface Plasmon Resonance (SPR): (a) Calculation of the dissociation constant (K_D) of the unmodified aptamer; (b) Calculation of the dissociation constant (K_D) of the modified biotinylated dUTPs aptamer. The aptamers were prepared in binding buffer (10 mM Phosphate buffer, 135 mM NaCl, 2.5 mM KCl, 1.5 mM $MgCl_2$ pH 7.4) ranging from 125 nM to 0 nM using 1 in 2 dilutions. The binding affinity of the aptamers was determined by BIAevaluation software using a 1:1 Langmuir binding model. The signal of the specific flow cell (with immobilised β-conglutin) was corrected by the subtraction of the signal from the control flow cell (activated with EDC/NHS and blocked with ethanolamine, but without β-conglutin immobilisation). The signals were also corrected via subtraction of the buffer signal obtained.

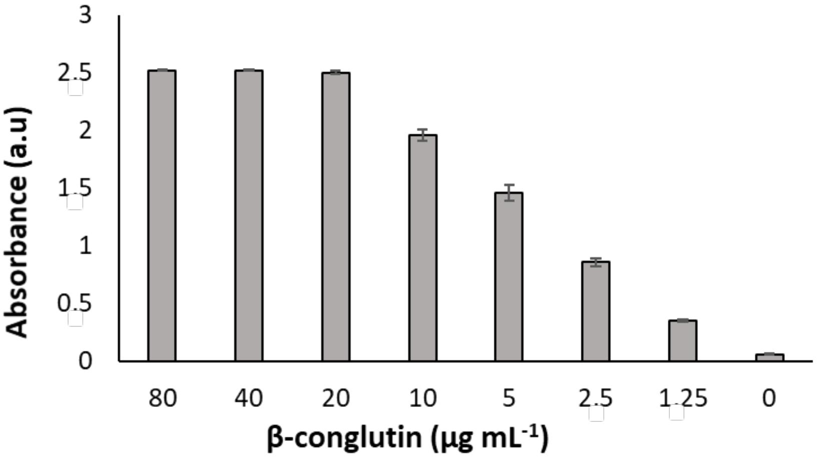

Figure S3. β-conglutin coating optimisation. Different concentrations of immobilised β-conglutin (80 – 0 μg mL^{-1}) in 50 mM carbonate-bicarbonate buffer pH 9.6 were incubated with a constant amount of biotinylated aptamer (1 nM) prepared in binding buffer (10 mM Phosphate buffer, 135 mM NaCl, 2.5 mM KCl, 1.5 mM $MgCl_2$ pH 7.4) followed by the addition of SA-polyHRP (dil. 1 in 20000 of 1 mg mL^{-1}). The absorbances were read at 450 nm after the wells being stopped with 1 M H_2SO_4 5 min following addition of TMB.

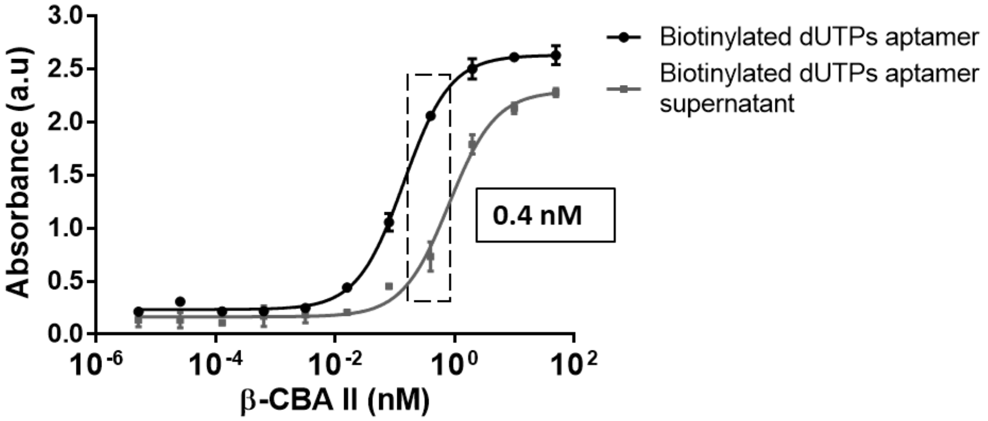

Figure S4. Optimal amount of modified aptamer. Different concentrations of aptamer (50 – 0 nM) were added to the wells of a microtitre plate modified with 20 μg mL^{-1} of β-conglutin (biotinylated dUTPs aptamer). The supernatant containing any unbound aptamer was removed and added to a fresh plate, also modified with 20 μg mL^{-1} of β-conglutin (biotinylated dUTPs aptamer supernatant). By comparing both binding curves, 0.4 nM aptamer was demonstrated to be optimal.

(a)

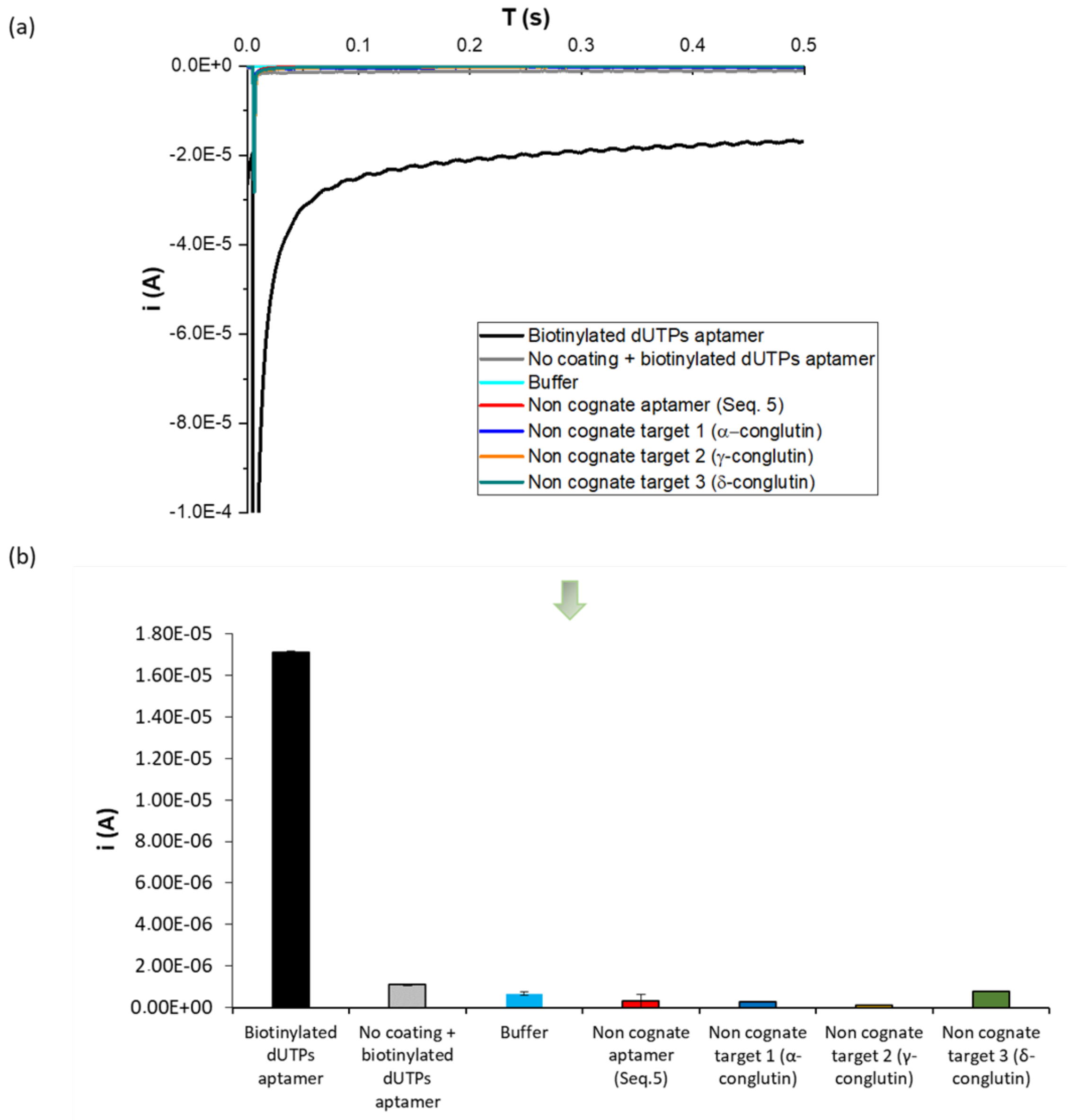

Figure S5. Evaluation of functionalised carbon screen-printed electrodes using fast chronoamperometry: (a) Signal obtained using fast chronoamperometry from 0 to 0.5 s; (b) Signal obtained at 0.5 s. All the aptamers (biotinylated dUTPs aptamer or non-cognate aptamer with biotinylated dUTPs) were prepared in binding buffer with a final concentration of 0.4 nM. Controls included: no coating (i.e. bare electrode), buffer instead of the aptamer, a non-cognate aptamer, and three non-cognate targets.

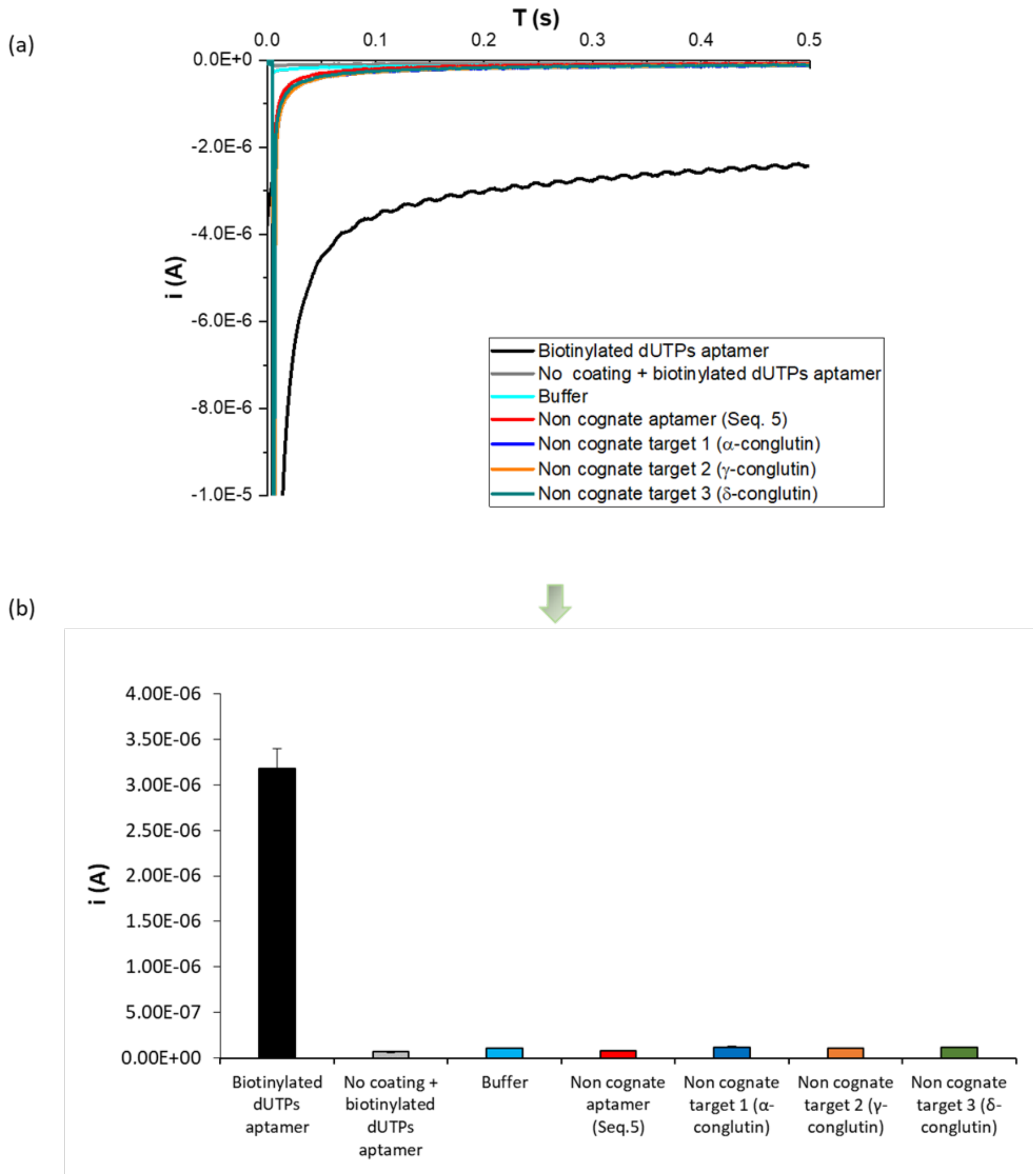

Figure S6. Evaluation of functionalised gold screen-printed electrodes using fast chronoamperometry: (a) Signal obtained using fast chronoamperometry from 0 to 0.5 s; (b) Signal obtained at 0.5 s. All the aptamers (biotinylated dUTPs aptamer or non-cognate aptamer with biotinylated dUTPs) were prepared in binding buffer with a final concentration of 0.4 nM. Controls included: no coating (i.e. bare electrode), buffer instead of the aptamer, a non-cognate aptamer, and three different non cognate targets.
